# Molecular predictors of response to tyrosine kinase inhibitors in patients with Non-Small-Cell Lung Cancer

**DOI:** 10.1186/1756-9966-31-77

**Published:** 2012-09-19

**Authors:** Samuel Murray, Vasilios Karavasilis, Mattheos Bobos, Evangelia Razis, Savvas Papadopoulos, Christos Christodoulou, Paris Kosmidis, George Fountzilas

**Affiliations:** 1 BioMarker Solutions Ltd. London, UK; & Department of Molecular Oncology, GeneKOR, Athens, Greece; 2 Department of Medical Oncology, “Papageorgiou” Hospital, Aristotle University of Thessaloniki School of Medicine, Thessaloniki, Greece; 3 Department of Pathology, Aristotle University of Thessaloniki School of Medicine, Thessaloniki, Greece; 4 First Department of Medical Oncology, “Hygeia” Hospital, Athens, Greece; 5 Department of Pathology, “Hygeia” Hospital, Athens, Greece; 6 Second Department of Medical Oncology, “Metropolitan” Hospital, Athens, Greece; 7 Second Department of Medical Oncology, “Hygeia” Hospital, Athens, Greece

**Keywords:** Predictive, Somatic mutation, EGFR, Gefitinib, Erlotinib, Response

## Abstract

**Introduction:**

Epidermal growth factor receptor (EGFR) tyrosine kinase inhibitors (TKIs) have become a treatment option in non-small-cell lung cancer (NSCLC) patients. However, despite their use in this disease, a significant number of patients will eventually develop resistance and relapse. In this study, we aimed to characterize several molecular events involved in potential resistance mechanisms to anti-EGFR treatment and correlate our findings with clinical outcome.

**Material and methods:**

The medical records of patients with NSCLC who received anti-EGFR TKIs in any line within the participating centers were reviewed and available paraffin embedded tissue was retrieved. Mutational analysis for *EGFR*, *KRAS, BRAF* and intron-exon 14 deletions of *MET*; FISH analysis for chromosomal gain or amplification for *EGFR*, *MET* and the deletion marker D7S486 were performed. Furthermore, the expression of EGFR and MET were analysed by immunohistochemistry. All results were correlated with treatment outcomes.

**Results:**

Between 10/2001 and 12/2009 from an initial cohort of 72 treated patients, 59 cases (28 gefitinib/ 31 erlotinib) were included in the analysis. The majority had adenocarcinoma histology (68%), and received treatment in the second line setting (56%). Disease control rate (DCR) was 25.4% for all patients. *EGFR* and *RAS* mutational rates were 33% and 10% respectively, no other mutations were identified. High EGFR expressing tumors were found in 7 of 45 cases and pEGFR positivity (IHC) was found in 56% of the cases; MET expression was found in 48% of tumors. *EGFR* gene amplification was found in 4 cases, two cases showed high polysomy; overall, 13% cases were FISH positive for *EGFR*. High polysomy of *MET* gene was detected in 1/43 cases tested. D7S486 locus deletion was detected in 15/37 (40%) of cases. *EGFR* mutational status and gene gain were both associated with more favorable DCR. No other associations between examined biomarkers and DCR or survival were noted.

**Conclusions:**

*EGFR* mutational status is a predictor for disease control in patients with NSCLC treated with anti-EGFR TKIs. The predictive role of several other molecules involved in potential resistance to anti-EGFR TKIs is worthy of additional investigation.

## Introduction

Non-small-cell lung cancer (NSCLC) has become the leading cause of cancer-related death in Western countries where the majority of patients present with advanced or metastatic disease [1]. The overall poor prognosis and the plateau of improvement in response and survival outcomes seen with chemotherapy over the last two decades, highlight the need for additional therapeutic strategies [2]. Over the last few years epidermal growth factor receptor (EGFR) has been identified as a promising therapeutic target due to its correlation with adverse disease characteristics such as advanced stage at diagnosis, and resistance to treatment [3-5].

Erlotinib (Tarceva®, OSI-Pharmaceuticals, New York, NY) was approved as mono-therapy in the second-third-line treatment of lung cancer [6]. This tyrosine kinase inhibitor (TKI) along with gefitinib (Iressa®, AstraZeneca, Macclesfield, UK) reversibly bind the ATP-binding pocket of the EGFR tyrosine kinase domain, thereby inhibiting auto-phosphorylation and stimulation of downstream signaling pathways resulting in inhibition of proliferation, delayed cell cycle progression, and increased apoptosis [7-11]. The more recent understanding that both of these agents display extremely high response rates in patients harboring somatic mutations in *EGFR* has resulted in the first molecularly stratified licensing approval for a drug in NSCLC [12]. Subsequent to the recent publication of the IPASS study, gefitinib was awarded license for the treatment of first line, chemotherapy naive advanced or metastatic patients with NSCLC based upon molecular stratification for the presence of activating somatic *EGFR* mutations [13].

Somatic mutations in the *EGFR* tyrosine kinase domain are correlated with improved response rates with both of these agents [14]. However, this is not the only biomarker correlated with response, *EGFR* gene gain is also a well characterised biomarker of TKI response [15], and there is evidence of co-segregation of mutation and gene gain [1,16]. Other predictive biomarkers have also been identified including a biomarker of non-responsiveness, somatic mutations in *KRAS;* these are also known to be mutually exclusive from *EGFR*[17]. Moreover, there are a number of patients who either do not respond in the presence of known predictive biomarkers, or who develop resistance to anti-EGFR TKIs. Several of the candidate biomarkers of either ‘acquired’ or ‘*de-novo*’ resistance to TKI treatment include secondary *EGFR* mutations (including T790M), and *cMET* gene gain [18]. In this retrospective clinical – translational study we aimed to characterise several of these molecular events and correlate them with response and outcome of patients treated with either of the EGFR TKIs.

## Patients and methods

### Patient’s selection

The medical records of all patients with histologically confirmed advanced or metastatic NSCLC treated within Hellenic Cooperative Oncology Group (HeCoG) participating centers, October 2001-December 2009, were retrospectively reviewed. Cases who received anti-EGFR TKI treatment were retrieved.

### Anti-EGFR treatment

Anti-EGFR treatment was introduced to NSCLC patients who had clinical stage IIIB, stage IV, or recurrent disease, and a measurable indicator lesion by RECIST classification that had not been irradiated. Patients could have received any number of prior chemotherapy regimens and 3 weeks must have elapsed since prior chemotherapy. Eligible patients had Karnofsky performance status (PS) ≥60% or ECOG PS ≥2, sufficient bone marrow function and adequate liver and kidney function. Patients with brain metastases stable for >3 months were also candidates for such treatment. All patients’ signed informed consent before starting treatment.

Patients must have been treated with either single agent gefitinib or erlotinib. Availability of paraffin-embedded tissue sample at diagnosis was also classified as an entry criterion for this study. All patients signed informed consent for the use of biological materials for research purposes. The study was conducted according to the Declaration of Helsinki and the guidelines for Good Clinical Practice. The bioethics Committee of Metropolitan Hospital approved the study and the collection of biological material.

### Patient evaluation and treatment

All patients received gefitinib at 250 mg per day orally or erlotinib at 150 mg orally. Gefitinib was supplied free of charge by AstraZeneca as part of an international compassionate use program. Since 2005 erlotinib was nationally approved for the treatment of NSCLC irrespective of *EGFR* mutational status. Treatment was administered daily with a treatment cycle constituting 28 days. Treatment was discontinued for up to 7 days for grade 3–4 toxicity, until resolution of toxicity to ≤1. For non-resolving toxicities of more than 15 days, treatment was ceased. Treatment was continued until disease progression, serious adverse toxicity, at the decision of the treating physician, or following voluntary patient withdrawal.

Patients were eligible for response evaluation after completion of >2 months treatment. Clinical data including smoking history, clinical stage, pathological diagnosis and response data for all patients was retrieved from their medical reports.

### Somatic mutation analyses

Genomic DNA was extracted from paraffin embedded tumors obtained retrospectively from HeCOGs Tumor Repository Bank, as previously described. All paraffin blocks were examined on H&E for histological verification according to W.H.O [19]. Tumors with >75% neoplastic cell content (%NCC) were considered as eligible for analysis. For biopsies with inadequate %NCC, macro-dissection on 5 μm sections was performed to increase the content to >75%. Mutational analysis for all genes was conducted as previously described [20]. The primer sequences for all reactions are available upon request. All studied exons were confirmed, for *EGFR*. Sequences were analyzed by BLAST and chromatograms by manual review, and compared to the following representative gene accession numbers: *EGFR*, NM_005228 and/or the *EGFR* gene sequence Accession number:AF288738; *KRAS*, gi:14277199 ( http://www.ncbi.nlm.nci).

The *EGFR* exon 21 L858R mutation [21] was also analyzed by PCR-RFLP based on the presence of a new *Sau*96I restriction site. Codons 12/13 of *KRAS* were also analyzed by PCR-RFLP [22,23]. *BRAF* exons 14 & 15 were analyzed as previously described [20], and the 3’ and 5’ intron-exon splice sites of *MET* exon 14 were also screened.

### Immunohistochemistry

**Figure 1:**
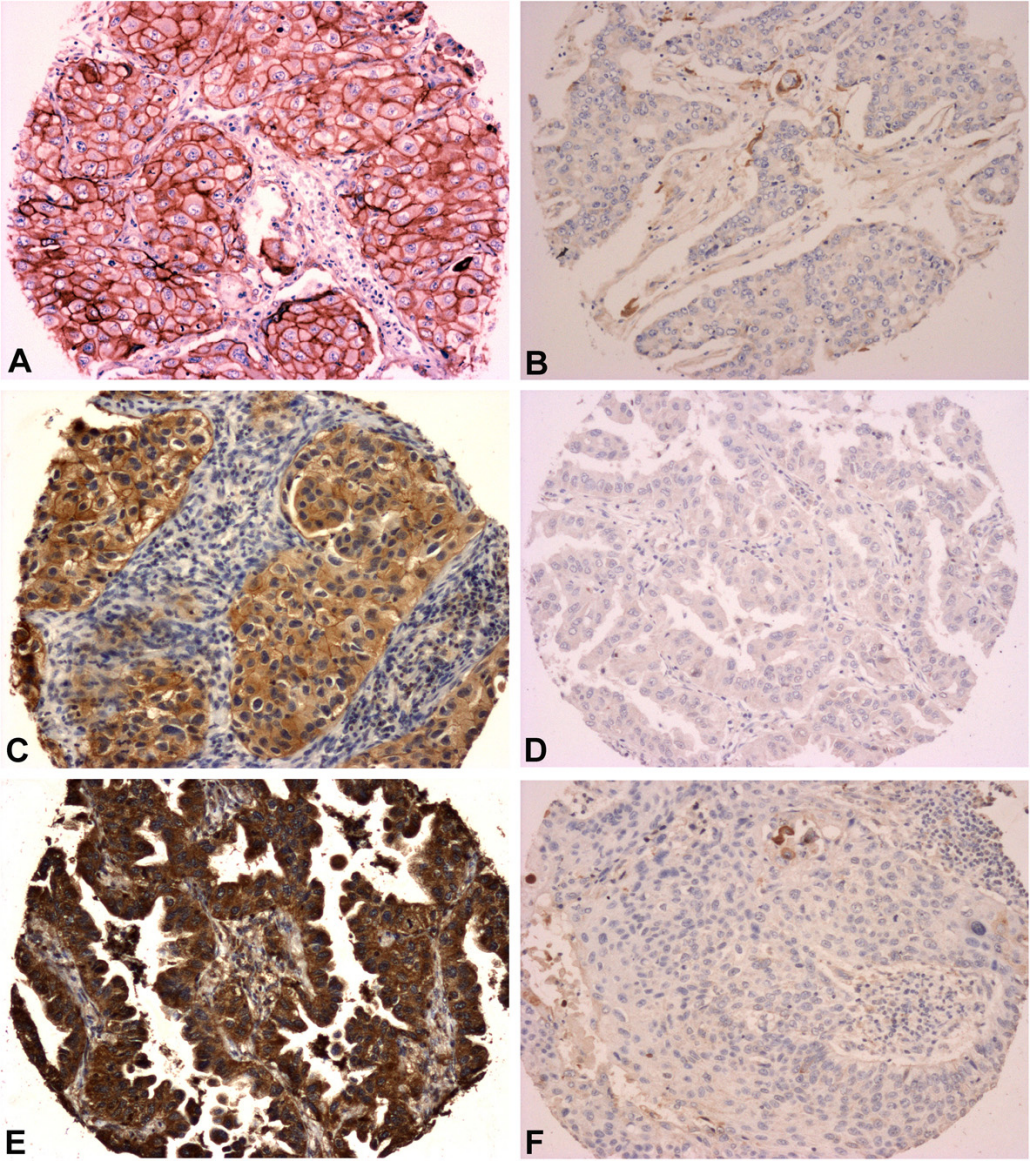
** Expression of proteins in NSCLC tumors studied by immunohistochemistry in tissue microarrays.A**) EGFR strong membrane positivity; **B**) EGFR absence; **C**) pEGFR^Tyr1173^ membrane and cytoplasmic positivity; **D**) pEGFR^Tyr1173^ lack of immunoreaction; **E**) c-MET strong cytoplasmic staining; **F**) Absence of c-MET staining. (Full size images X100).

Following H&E review by an experienced pathologist, only the cases with adequate material were selected for further analysis (Figure 1). Tissue microarrays (5x 0.6 mm diameter cores per case), were created. Cases not included on TMAs were further handled as whole tissue sections. Immunohistochemical staining was performed according to standard protocols, with slight modifications, on serial 2.5 μm thick sections using the Bond Max^TM^ (Leica Microsystems, Germany/Menarini Diagnostics, Athens, Hellas) and i6000 (Biogenex, USA) auto-stainers. The conditions of staining for the antibodies against EGFR (clone 31 G7, Invitrogen, CA, USA) and c-MET/HGFR (8 F1, Novocastra^TM^, Leica Biosystems, UK) were as previously described [24]. For the detection of phosphorylated EGF Receptor at Tyr1173 monoclonal rabbit antibody (clone 53A5 CST, MA, USA) at a dilution of 1:150 was used, staining was performed using a Bond Max autostainer. 

### Immunohistochemical scoring

EGFR protein staining was evaluated, using a previously proposed semi-quantitative approach based on staining intensity (0–4) and percentage of stained tumor cells (0–100) [25]. Diffuse cytoplasmic or granular staining was diagnosed as negative. Scores of 0–200 were considered as negative/low expression, scores of 201–400 were considered as positive/high expression. For evaluation of phospho-EGFR^Tyr1173^ and c-MET expression we used a semi-quantitative scoring system based on intensity and staining pattern. Intensity was scored as follows: 0 = no staining, 1 = weakly, 2 = moderately, and 3 = strongly positive. The scoring of the staining pattern was based on the percentage of positive tumor cells: 0 = 0 to 5%, 1 = 6 to 25%, 2 = 26% to 50%, 3 = 51% to 100%. The localization of staining for each protein was also indicated, as cytoplasmic and cytoplasmic/membranous for MET and nuclear, cytoplasmic and cytoplasmic/nuclear for phospho-EGFR^Tyr1173^. The total score was calculated as the sum of the intensity score and the staining pattern score. Cases with a total score at least 2, were considered as positive, whereas cases with a total score of 0–1 were grouped together and considered as negative or low-expressing tumors.

### FISH

FISH was performed on 4.5 μm TMA sections or whole FFPE archival tissue samples using the ZytoLight ® SPEC *EGFR/CEN* 7 Dual color probe (ZytoVision, Bremerhaven, Germany/Menarini Diagnostics, Greece), the LSI *D7S486/CEP7* Dual Color Probe, (Abbott Molecular, IL, USA) and the specific *HGFR/MET* gene at region 7q31, Poseidon™ Repeat Free™ *MET/SE7* probe (Kreatech Diagnostics, NL) as previously described [26]. FISH assays, were captured by a computer-controlled digital camera and processed by commercially available software (XCyto-Gen, Alphelys, France). Sequential, digital images were captured by a stack motor for each fluorescence filter and the resulting images were reconstructed with blue, green and orange or red pseudo-colors. Sixty non-overlapping intact nuclei from the invasive part of the tumor were evaluated for each case according to morphological criteria using DAPI staining.

FISH patterns for the *EGFR* gene were defined as previously described [27]. *MET* gene status was classified according to Cappuzzo et al. [28] in two strata as follows: 1) FISH positive if mean *MET* gene ratio was ≥5 gene copies per cell, 2) FISH negative if mean *MET* gene ratio was <5 gene copies per cell. The status of the *D7S486* locus was evaluated as follows: amplification if the ratio *D7S486/CEP7* was ≥2, and deletion if ratio was <0.7.

### Statistical analysis

Endpoints included PFS (progression free survival) and overall survival (OS) in association with the candidate biomarkers. PFS was computed as the time from initiation of treatment until recurrence of tumor or death from any cause. Survival was defined as the time from first day of treatment until death from any cause. Disease control rate (DCR), was defined as the sum of patients who achieved complete (CR) or partial response (PR) and those who had stability of their disease (SD). Fisher’s exact test was used for comparing groups of categorical data, while for continuous data the Mann–Whitney test was used. P values of at least 0.05 were considered statistically significant. Kaplan-Meier curves and log-rank test were used for comparing time to event distributions. Univariate Cox regression analyses were performed to estimate hazard ratios (HR). All analyses were performed using SPSS version 15.0, in the HeCOG data office.

## Results

A total of 72 patients received treatment. However, 8 cases were excluded due to incomplete medical records and a further 5 due to insufficient tumor in their biopsies. Baseline characteristics of the 59 eligible cases for the translational study (28 gefitinib, 31 erlotinib) are listed in Table 1. Adenocarcinoma was identified in the majority of cases (68%). Approximately two thirds of patients were males, and 32% had never smoked. There were no significant differences in selected patients and tumor characteristics between the two treatment groups.

**Table 1:** Selected patient and tumor characteristics

		**Gefitinib N = 28**		**Erlotinib N = 31**	
Age (at diagnosis)	Median (range)	63.5 (37–80)		65.8 (34–79)	
		**N**	**%**	**N**	**%**
Gender	Male	20	71	20	65
	Female	8	29	11	35
Smoker	No	7	25	12	39
	Yes	20	71	16	52
	Unknown	1	4	3	10
Histology	Squamous cell Ca	4	14	3	10
	Adenocarcinoma	18	64	22	71
	Large cell Ca	1	4	-	-
	Mixed type Ca	1	4	1	3
	Undifferentiated/Unclassified Ca	3	11	2	6
	Unknown	1	4	3	10
Grade	1	3	11	2	6
	2	4	14	7	23
	3	6	21	6	19
	Undifferentiated	3	11	-	-
	Unknown	12	43	16	52
Performance status	0	6	21	7	23
	1	15	54	16	52
	2	-	-	1	3
	Unknown	7	25	7	23
Line of TKI treatment	1st	4	14	9	29
	2nd	18	64	15	48
	>2nd	6	21	7	22

Anti-EGFR treatment was given as first line treatment in 13 patients, 33 patients were treated in the second line setting and a further 13 patients received anti-EGFR treatment as third or fourth line. Treatment resulted in a DCR of 25.4% (5 PR, 1 gefitinib). Twelve patients were not evaluable for response, and 2 had early disease related death.

After a median follow up of 40 months, 51 patients progressed and 49 died (26 gefitinib). Median PFS was 3.4 months (95% Confidence interval [CI]: 2.7-4.1) and median survival 11 months (95% CI: 6.5-15.6). There was a significant difference in PFS between the patients receiving a TKI in the first and second (or greater) line of treatment (12.6 [95%CI: 3.6-21.5], 3.2 [95%CI: 2.8-3.6] months, log-rank p = 0.037, 1^st^ line versus ≥2 lines). Overall survival was also greater in patients treated in the 1^st^ line versus subsequent lines (23.9 [95%CI: 19.8-27.9] versus 8.6 [95%CI: 1.9-15.2] months; log-rank p = 0.012).

### *EGFR*, *KRAS*, *BRAF* and *MET* mutational analysis

*EGFR* mutational analysis was performed in 33 cases. In 11 cases *EGFR* mutations were found. Mutational analysis for *KRAS* demonstrated 3 cases with mutant *KRAS*, of 30 cases tested, (Table 2). *EGFR* and *KRAS* mutations were mutually exclusive. Characteristics of the *EGFR* mutation positive patients are shown in Table 3. No mutations in *BRAF* and no intron-exon 14 deletion of the *MET* gene were identified.

**Table 2:** Biomarker evaluation in total population and according to treatment

		**Total**		**Gefitinib**		**Erlotinib**	
		**N**	**%**	**N**	**%**	**N**	**%**
**Gene mutation status**							
*KRAS* (N = 30)	WT	27	90	21	88	6	100
	Mutated	3	10	3	13	0	0
*EGFR* (N = 33)	WT	22	67	20	74	2	33
	Mutated	11	33	7	26	4	67
**IHC**							
EGFR (HIRSCH) (N = 45)	Negative	38	84	14	93	24	80
	Positive	7	16	1	7	6	20
pEGFR (N = 43)	Negative	19	44	7	47	12	43
	Positive	24	56	8	53	16	57
CMET (N = 42)	Negative	22	52	5	36	17	61
	Positive	20	48	9	64	11	39
**FISH**							
*EGFR* (N = 45)	Negative	39	87	16	100	23	79
	High polysomy	2	4	0	0	2	7
	Amplified	4	9	0	0	4	14
*EGFR* (N = 45)	Negative	39	87	16	100	23	79
	Positive	6	13	0	0	6	21
D7S486 (N = 37)	Deletion	15	40	8	50	7	33
	Normal	22	60	8	50	14	67
*MET* (N = 43)	Negative	42	98	14	93	28	100
	Positive	1	2	1	7	0	0

**Table 3:** **Selected clinicopathological characteristics, gene profiling and survival data for patients harboring *EGFR*mutations **

	**Age**	**Gender**	**Histology**	**Smoking status**	**Treatment Line**	**cMET (IHC)**	**EGFR (HIRSCH)**	***EGFR*(FISH)**	***MET*(FISH)**	***D7S486*(FISH)**	**Best Response**	**PFS (months)**	**Survival (months)**
1	37	Female	AdenoCa	Smoker	1st	ND	ND	ND	ND	ND	SD	12.59	25.48
2	56	Male	AdenoCa	Smoker	4th	ND	ND	ND	ND	ND	PD	2.39	4.23
3	76	Male	Squamous	Smoker	2nd	ND	ND	ND	ND	Deletion	PR	11.67+	11.67+
4	64	Male	AdenoCa	Non-smoker	2nd	Negative	Negative	Negative	Negative	Deletion	NE	8.52	29.51
5	76	Female	AdenoCa	Non-smoker	1st	Negative	Negative	ND	Negative	Normal	SD	12.69	23.38
6	78	Female	AdenoCa	Non-smoker	1st	Negative	Negative	Negative	Negative	Normal	PR	20.52	21.34
7	67	Male	AdenoCa	Smoker	2nd	Negative	Negative	Negative	Negative	Normal	PD	3.25	28.49
8	62	Female	AdenoCa	Non-smoker	1st	Positive	Positive	Positive	Negative	Normal	SD	40.20+	40.20+
9	47	Male	AdenoCa	Smoker	2nd	ND	ND	ND	ND	ND	NE	4.00	4.00
10	43	Female	AdenoCa	Non-smoker	2nd	ND	ND	ND	ND	ND	PD	2.56	2.85
11	63	Male	Squamous	Smoker	2nd	ND	ND	ND	ND	ND	PD	2.26	12.49

ND: not done; NE: non-evaluable.

### Protein expression analysis (Immunohistochemistry)

High EGFR expressing tumors were found in 7/45 tested cases, 1/15 from the gefitinib treated group and 6/30 from the erlotinib group. Phospho-EGFR^Tyr1173^ positivity was found in 24 (56%) cases, with similar results in tumors from the patient treatment groups (53% for the gefitinib treated group and 57% for the erlotinib group). c-MET expression was found in nearly half of tested tumors (20/42, 48%). (Figure 1 and Table 2)

### *EGFR*, D7S486 and *MET* FISH analysis

**Figure 2:**
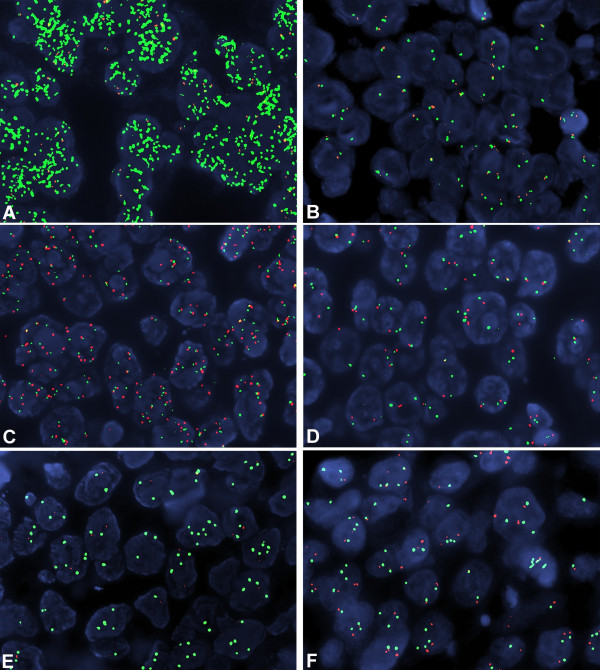
** Fluorescence in situ hybridization with gene, locus and centromeric specific probes.A**) Neoplastic nuclei showing *EGFR* gene amplification (green signals) and polysomy of chromosome 7 (CEP7-orange signals); **B**) Representative case with normal *EGFR* gene status; **C**) *MET* high level gain (red signals) accompanied by high polysomy of chromosome 7 (CEP7-green signals); **D**) Normal *ME*T gene status, **E**) D7S486 locus deletion (red signals); **F**) D7S486 locus normal status. (Full size images X1000).

*EGFR* gene amplification was found in 4 cases. Two cases showed high polysomy (≥ four copies of the gene in ≥ 40% of cells) and overall, 6/45 (13%) cases were considered as FISH positive. High polysomy of *MET* gene was detected in 1/43 cases tested. Six cases showed mean copy number of *MET* gene from 3.11 to 4.05 and were considered as cases with low gain. D7S486 locus deletion was detected in 15/37 (40%) of cases; amplification of the locus was not found in our cohort. (Figure 2 and Table 2)

### Correlation of biomarkers with clinical outcome

*EGFR* mutation and FISH status were both associated with DCR. Patients, whose tumors had an *EGFR* mutation, had a DCR of 45.5% (5/11 patients), whereas among 22 wild type tumors, DCR was observed in only one patient (p = 0.01). Patients with high polysomy and amplification of *EGFR* gene (n = 6), considered as FISH positive, showed a higher DCR compared with patients with *EGFR* FISH negative tumors (66.7% versus 12.8%). No other associations between examined biomarkers and DCR were noted (Table 4).

**Table 4:** Association of disease control rate (DCR) with examined biomarkers

		**Response**		
		**Disease control**	**Disease progression**	**p-value**
		**N (%)**	**N (%)**	
**Gene status**				
*KRAS* (N=30)	WT	7 (0)	20 (87)	0.999
	Mutated	0 (0)	3 (13)	
*EGFR* (N=33)	WT	1 (17)	21 (78)	0.010
	Mutated	5 (83)	6 (22)	
**IHC**				
EGFR (HIRSCH) (N=45)	Negative	8 (73)	30 (88)	0.337
	Positive	3 (27)	4 (12)	
pEGFR (N=43)	Negative	4 (36)	15 (47)	0.728
	Positive	7 (64)	17 (53)	
cMET (N=42)	Negative	5 (50)	17 (53)	0.999
	Positive	5 (50)	15 (47)	
**FISH**				
*EGFR* (N=45)	Negative	5 (56)	34 (94)	0.005
	High polysomy	2 (22)	0 (0)	
	Amplified	2 (22)	2 (6)	
*EGFR* (N=45)	Negative	5 (56)	34 (94)	0.010
	Positive	4 (44)	2 (6)	
*D7S486* (N=37)	Deletion	3 (43)	12 (40)	0.999
	Normal	4 (57)	18 (60)	
*MET* (N=43)	Negative	11 (100)	31 (97)	0.999
	Positive	0 (0)	1 (3)	

Univariate Cox regression analyses, adjusted for chemotherapy agent, revealed that only *KRAS* mutations were associated with shorter survival (HR: 6.2, 95% CI: 1.6-24.6, p = 0.009). No other association was found among the remaining biomarkers and survival parameters.

## Discussion

Although EGFR-targeted therapies have demonstrated activity in unselected NSCLC patient populations, it is likely that these agents will be most effective in select subpopulations. Asian ethnicity, female gender, nonsmoking history, and adenocarcinoma histology were associated with better responsiveness to EGFR TKIs in several clinical studies. Furthermore, several molecular characteristics have been associated with either better responsiveness or resistance to EGFR-targeted agents. However, there are different ways of testing for EGFR, including somatic mutation testing, IHC, and FISH. Although previously published data did not use a standardized approach, large prospective, randomized trials are ongoing assisting in the validation of such testing.

In our study 11% of patients tested positive for *EGFR* FISH (gene amplification/high polysomy), which was only correlated with an improved PFS. *EGFR* gene amplification analysed by FISH has not consistently been demonstrated to be a predictive biomarker of response [13]. In the BR.21 trial, patients with high polysomy/amplification were found to have a significantly higher RR than patients without these tumor qualities, and *EGFR* gene amplification was predictive of a survival benefit with erlotinib. Similarly, results from the ISEL trial showed a greater survival benefit with gefitinib among patients with high *EGFR* gene copy number, compared with patients who had a low *EGFR* gene copy number (GCN). Both PFS and survival were significantly longer among patients who were *EGFR* FISH positive than among patients who were *EGFR* FISH negative [29,30]. Conversely, patients with a high *EGFR* GCN by FISH did not demonstrate a survival advantage with gefitinib over docetaxel in the second-line setting in a trial specifically designed to investigate GCN effect, the INTEREST trial [31]. Our recent meta-analysis of the predictive ability of GCN indicated that it is a fairly good biomarker for response [14], however, only in non-Asian patient populations was it shown to be predictive of improved PFS and OS, albeit from a limited number of studies most of which were not designed to investigate the particular biomarker [15]. Our data correlates with these previous data sets but does not assist greatly in understanding the differences seen between “Asian” and “non-Asian” studies.

Regarding IHC expression of EGFR, this was found positive in 16% of the cases tested and no correlation with clinical outcome was demonstrated. The IHC expression of EGFR protein varies across several studies and as such, has been an inconsistent predictor of response to EGFR inhibitors. In a retrospective analysis of tumor biopsy samples from patients treated in the BR.21 trial, 57% were found to over-express EGFR by IHC. Response to EGFR agonists was found higher among patients expressing EGFR, though the difference was statistically insignificant. Furthermore, EGFR protein status was not an independent predictor of OS in this study. In opposition, in the ISEL trial, patients with EGFR expressing tumors, as detected by IHC, had significantly longer OS than patients with EGFR negative tumors. A combination of IHC and FISH status may be an effective predictor of responsiveness to EGFR TKIs, however, in our study this was not feasible due to the small number of cases for EGFR FISH and IHC.

It has been demonstrated that somatic mutations in the *EGFR* TK domain are associated with responsiveness to EGFR TKIs [14]. We found that patients harboring *EGFR* mutations in exon 19/21 had a significantly better DCR as compared with those with no detectable mutations. These patients had also a longer PFS. Data from the INTEREST trial also showed that *EGFR* mutation was a predictive marker of prolonged PFS. More recently, the phase III IPASS study that randomized 1,217 patients to gefitinib versus carboplatin plus paclitaxel indicated the superior benefit obtained with gefitinib restricted to the *EGFR* mutation positive population. Several subsequent studies support this data [32,33].

Although treatment with EGFR TKIs provides clinical benefit to some patients, many are primarily resistant to treatment. Furthermore, virtually all patients with an initial response to TKIs, even in the presence of activating sensitizing mutations, eventually relapse and demonstrate TKI resistance. Multiple underlying mechanisms of resistance have been described, including *EGFR* mutations, the phosphatase and tensin homologue deleted on chromosome 10 (PTEN) pathway, *MET* amplification, and *KRAS* mutations [18].

Whereas activating mutations in the EGFR TK domain are associated with greater sensitivity to TKIs, some mutations are associated with resistance. A secondary mutation of *EGFR* at exon 20, T790M has been identified in as many as 50% of patients who stop responding, and is thought to account for most cases of acquired resistance. Nevertheless such mutations were not identified in our study. Re-biopsy following relapse was not conducted in this study limiting our understanding of the possible acquisition of T790M. Other *EGFR* mutations reportedly correlated to resistance, such as D761Y, L747S, and A854A, were also not identified in our series.

Preclinical data suggest that amplification of the *MET* proto-oncogene may play a role in acquired resistance to EGFR TKIs through the PI3K pathway. *MET* amplification has been detected in lung cancer cell lines that have acquired resistance to gefitinib. Current evidence implies that *MET* amplification occurs independently of T790M and it has been proposed that concurrent inhibition of both may further improve clinical outcomes. Recently, a large retrospective study of surgically resected NSCLC showed that increased *MET* GCN is an independent negative prognostic factor [28]. In our small series, high MET gene gain was found in only one patient, and overall gene gain in 16% of cases. None of the tested cases showed amplification. Previous reports, using different interpretation methodologies of *MET* gene status, showed a gene gain between 11-50%, and amplification in 3-11% of patient’s tumors [28,34].

Loss of heterozygosity (LOH) has been frequently detected at chromosome 7q31 region in several solid tumors including head and neck squamous cell carcinomas, prostate, breast and ovarian cancers, suggesting the existence of tumor suppressor genes. Deletions at 7q31 region appear to be very common phenomenon in cancer, and are correlated with a more aggressive phenotype. Monosomy 7 and loss of chromosome 7q are also observed in myelodysplastic syndromes (MDS) and acute myeloid leukemia (AML). In some instances, these abnormalities are associated with patient outcome. D7S486 locus deletion has been frequently detected in head and neck squamous cell carcinomas and prostate adenocarcinomas and has been associated with higher grade and advanced tumor stage [35]. In our study D7S486 locus deletion was detected in 40% of cases but no association with clinical outcome was demonstrated. Nevertheless, the role of LOH at 7q31 region has not been investigated in NSCLC and neither its possible associations with *MET* gene, which is mapped to 7q31 seems to be an interesting area of investigation in NSCLC.

KRAS is a signaling molecule downstream of EGFR. KRAS and EGFR play pivotal roles in the development and growth of NSCLC, especially in patients with adenocarcinoma histology. Patients with *KRAS* mutations respond poorly to EGFR inhibitors, with increasing data implicating *KRAS* mutations as a mechanism of primary resistance to EGFR TKIs [17]. Activating mutations in codons 12 and 13 of the *KRAS* gene are present in approximately 15–30% of NSCLC cases [36]. Only 10% of our studied cases were *KRAS* mutation positive, they were mutually exclusive from *EGFR* mutations, and none of them responded to treatment. This correlates with our previous analysis [17].

This study has several limitations. It is retrospective in nature, with significant patient heterogeneity, includes only a small number of cases, and not all specimens were appropriate for molecular analysis (a common finding in several NSCLC studies [12]). We have also combined patients treated with gefitinib and erlotinib. Despite these limitations *EGFR* status was once again demonstrated to be a predictor for disease control and PFS, and KRAS a poor predictive marker. Although our study did not identify any other provisional candidate biomarker of response or resistance, due to the small size of the study and the inevitable relapse of virtually all patients it is now time to investigate, in a prospective manner, the role of several biomarkers of acquired and de-novo resistance in light of the routine clinical testing for *EGFR* status.

## Competing interests

Consultant or Advisory role: Dr. S. Murray, Merck KGaA, Darmstadt, Germany. Merck distribute the MoAb Cetuximab (ERBITUX®); AstraZeneca, Maccelsfield, United Kingdom. AstraZeneca are proprietors of Iressa® (gefitinib); Amgen Thousand Oaks Ca, USA. Amgen distribute the MoAb Panitumumab (Vectibix®). Professor G. Fountzilas, Pfizer Hellas, advisory role, Roche Hellas commercial research grant, Genesis – Pharma, Hellas. No other author declares a conflict of interest.

## Supported by a Hellenic Cooperative Oncology Group Research Grant (HE TRANS_02)

## Authors’ contributions

MB carried out the IHC and ISH studies; SP independently assessed the IHC and ISH studies; SM carried out the molecular genetic studies; all authors (SM, VK, MB, ER, SP, CC, PK, GF) participated in design of the study, analysis of the data, statistical analysis, and drafting of the manuscript. All authors read and approved the final manuscript.
